# Supplementary material for: Correlation of serum screening and neuroimaging studies: A comparative analysis for diagnostic evaluation of neurocysticercosis among people with epilepsy attending mental health clinics in selected district hospitals of Tanzania
Source: PLoS Negl Trop Dis. 2025 Apr 10;19(4):e0013002. doi: 10.1371/journal.pntd.0013002 (PMC12040232; doi:10.1371/journal.pntd.0013002)
Supplement: Attachment — Submitted filename: Letter with responses to reviewers_2.pdf [file pntd.0013002.s002.pdf]

Subject: Authors' response to reviewer comments

Manuscript Number: PNTD-D-24-01083

Title: Correlation of serum screening and neuroimaging studies: a comparative analysis for diagnostic evaluation of neurocysticercosis among people with epilepsy attending mental health clinics in selected district hospitals of Tanzania

Authors: Charles E. Makasi, Bernard Ngowi, Andrew Kilale, Godfrey Guga, Michael J. Mahande, Johnson Mshiu , Abisai Kisinda, Blandina T. Mmbaga

Journal: PLOS Neglected Tropical Diseases

Dear Editor,

Thank you for the invitation to resubmit our manuscript entitled: **"Correlation of serum screening and neuroimaging studies: a comparative analysis for diagnostic evaluation of neurocysticercosis among people with epilepsy attending mental health clinics in selected district hospitals of Tanzania"**

We appreciate the reviewers' useful comments and suggestions to improve our manuscript. We were able to address all the comments and suggestions, and therefore we are expecting that the manuscript can now be accepted in your journal. Please find our point-by-point responses to the reviewers' comments and concerns in the tables below:

A point-by-point response to reviewers' comments

**Comment**	**Response**
**Methods**<br>-Are the objectives of the study clearly articulated with a clear testable hypothesis stated?<br><br>-Is the study design appropriate to address the stated objectives?<br><br>-Is the population clearly described and appropriate for the hypothesis being tested?<br><br>-Is the sample size sufficient to ensure adequate power to address the hypothesis being tested?<br><br>-Were correct statistical analysis used to support conclusions?<br><br>-Are there concerns about ethical or regulatory requirements being met?	We thank the reviewers for reminding us of these key evaluation metrics, frankly speaking, our objectives have been well articulated fitting to the clear testable hypothesis. The design was appropriate for the stated objectives although there was a limited sample size due to the difficulty in availability of the participants and inadequately short study duration. However, there was a proper robust statistical analysis which led to the valid findings. Otherwise, there was a well-defined population. Overall, the study framework and methodological procedures were scientifically sound.
**Reviewer 1:** Aim of study	
The aim of the study has been mentioned; however, the author could specify the objectives of the study aligned with the conclusion. Although it's a part of a larger study, the author could write about the study design sample size calculation, and sampling method in detail. The statistical methods used were appropriate and ethical concerns were addressed, however, the author could share the sample of the participation information sheet to bring more clarity.	We thank the reviewer for this suggestion. While the aim of the study has been mentioned as stated (Line 136-138); we can explicitly specify the objectives as follows to align with the conclusion;<br>1. To assess the correlation between serum screening and neuroimaging studies among people with epilepsy.<br>2. To assess the diagnostic performance of serum cysticercosis tests using sensitivity, specificity, predictive values, likelihood ratios, and Receiver Operating Characteristic (ROC) analysis.<br>3. To evaluate the efficiency of the parallel testing methods to assess the diagnostic performance for the ruling out of the false negative results
**Reviewer 2:** Methodology<br>The methodology seems appropriate and well described, with most procedures clearly outlined.<br>However, some parts seem a bit confusing to me, due to repeated information. In line 226, it mentions the sample collection locations, providing the same information again in line 230	Thank you for identifying this redundancy. We have revised the text, deleted the repetition in line 230, and consolidated the sample collection details in line 226. We hope that this revision improves the clarity and flow of the methodological text.<br><br>The revised information from line 226 reads as follows;<br>"The collection of samples was conducted at Kongwa and Chunya district hospitals. The samples were

	shipped using a project vehicle to the analytical point at the NIMR-Mbeya laboratory with an escort of the laboratory expert ”.
**Reviewer #3:**<br>L94-95: Rephrase and emphasize on blood collection, volume, and anticoagulant….<br>The material and methods, result, and discussion sections need to be shorter. Please be more specific and avoid too many details. If methods have already been published, use citations. For commercial kits, use “following manufacturers procedure.”	Thank you for the feedback. When going through lines 94 and 95, we noticed that they do not contain the information mentioned for the collection of blood samples, volume, or anticoagulant. We would be glad to have the reviewer's clarification if the details are expected here.
**Results**<br>-Does the analysis presented match the analysis plan?<br><br>-Are the results clearly and completely presented?<br><br>-Are the figures (Tables, Images) of sufficient quality for clarity?	As stated in the previous key evaluation metrics, we still insist that the analysis was proper for the data and matched the analysis plan with results clearly and appropriately presented. The tables and figures are clear with sufficient quality
**Reviewer #1:** The analysis plan is appropriate, however, figure 1 and Table 1 data are missing from the result section. Likewise, Figure 2 and Table 4 data is not matching (It was mentioned 8 (22.2%) of 36 PWE who went for CT scan; (7 from the CC-positive group and one from PWE from CC negative) with neurological presentation showed NCC-typical lesions, But in Table 4 it was 10 with NCC). The author could correct the numbers where ever necessary.	Thank you for the observation. We declare that we encountered an error during the computation, which has now been corrected. Therefore, Tables 4, 5, 6, and Figure 3 have been updated to ensure consistency.
**Reviewer #2:** The results, although interesting, seem a little confusing to me in the way they are described. There is a lot of information that seems unnecessary to me, because it is in the table (i.e. p-values), and even highlighted in bold.<br>It would be interesting to show an image of the CT scan	Thank you for this valuable feedback and for highlighting areas to improve the clarity of our results.<br><br>We agree that some details such as p-values are presented in the tables and can be streamlined in the text for better readability. We have revised the results section to reduce redundancy and enhance clarity focusing on key findings.<br><br>In addition, we appreciate your suggestion to include an image of the CT scan. We are not sure if we’ll be allowed to show all the images of the CT scan in this manuscript; however, we are flexible to incorporate a representative CT scan image to visually support the findings if permitted by ethical and privacy

	considerations. We hope these changes have improved the presentation and flow of the results
**Reviewer #3:** The material and methods, result, and discussion sections need to be shortened, please be more specific, and avoid too many details. If methods have been already published use citation. For commercial kits use "following manufacturers procedure".	**Done:** CT scan examination methods referred to the published article by Makasi et al., 2024.<br><br>**Done:** Serological tests were done following the manufacturer's procedures.
**Conclusions**<br>-Are the conclusions supported by the data presented?<br><br>-Are the limitations of analysis clearly described?<br><br>-Do the authors discuss how these data can be helpful to advance our understanding of the topic under study?<br><br>-Is public health relevance addressed?	The conclusion has been amended following the reviewers' comments and the strength and limitations analysis of the study has been clearly described. Applicability of the data has been suggested with its public health relevance
**Reviewer #1:** The author could rephrase the conclusion aligned to the aim, i.e: to assess the correlation between serum screening and neuroimaging studies among PWE attending mental health clinics in selected district hospitals of Tanzania.<br>All other aspects have been mentioned clearly	Done: The conclusion has been improved as follows;<br>"In summary, this study demonstrated that parallel testing of the serum cysticercosis tests produced the proper validity of the tests. Multiple evaluation techniques, such as receiver operating characteristics curve, likelihood ratios, sensitivity and specificity, and positive and negative predictive values, are very crucial for the verification of the tests' ability to offer distinct perspectives on the test's efficacy. This robustness of a thorough approach increases confidence in the use of serum cysticercosis tests in our everyday practice, even though sensitivity and specificity may have been impacted by our biological sample processing. In every other case, PWEs had local access to uncommon diagnostic health services. Overall, this study provides essential evidence that can inform practical application as well as policy changes, marking a step forward in the field of T. solium neurocysticercosis diagnostic services."
**Reviewer #2:** The results are well discussed, in a well-organized way, and supported by a good list of bibliographic references, leading to a supported conclusion. It is worth highlighting the fact that it presents the strengths and limitations of the study, which I think is an advantage of the document.	Thank you for identifying this and we appreciate your supportive advice. We have highlighted this as one of the strengths of this document as recommended in the strength and limitations section of the article.

**Reviewer #3**: Conclusion need to be improved.	**Done:** Conclusion revised and improved
**Editorial and Data Presentation Modifications?**<br><br>Use this section for editorial suggestions as well as relatively minor modifications of existing data that would enhance clarity. If the only modifications needed are minor and/or editorial, you may wish to recommend "Minor Revision" or "Accept".	
**Reviewer #1:** Minor Revision	
**Reviewer #2:** Lines 101, 103, 104,105, 113, 114, 234 – (T.solium) should add space after "T." - T. solium<br>Line 103 – missed the "a" at "cellulosae and "italics – Cysticercus cellulosae<br>Line 232 – Should be T. solium<br>Line 549 - missed the italics – Helicobacter pylori<br>Lines 533, 538, 554, 559, 572, 597, 606, 619, 640, 645 – missed the italics – Taenia solium<br>Line 636 - missed the italics – Leishmania<br>Line 653 - missed the italics – Helicobacter pylori<br>Figure 1 - should review how to cite the source of the image	**Space added** after "T." (at T.solium) for Lines 101, 103, 104,105, 113, 114, 234 and all other areas for the entire article.<br><br>**Done:** Line 549 – done for the italics – *Helicobacter pylori*<br><br>**Done:** Lines 533, 538, 554, 559, 572, 597, 606, 619, 640, 645 – for the italics – *Taenia solium*<br>Done: Line 636 – for the italics – *Leishmania*<br>Done: Line 653 – for the italics – *Helicobacter pylori*<br><br>**Done:** Figure 1 – reviewed on how to cite the source of the image.<br>**BUT** Figure 2 has also been amended and some details added for clarity
**Reviewer #3:** (No Response)	
**Summary and General Comments**<br><br>Use this section to provide overall comments, and discuss strengths/weaknesses of the study, novelty, significance, general execution and scholarship. You may also include additional comments for the author, including concerns about dual publication, research ethics, or publication ethics. If requesting a major revision, please articulate the new experiments that are needed.	

**Reviewer #1:** Congratulations to the author for choosing a pertinent topic for research. Over all the quality of the article is satisfactory.	We highly appreciate for the complement
**Reviewer #2:** This paper is about the correlation of serum screening and neuroimaging in the diagnostic evaluation of neurocysticercosis among people with epilepsy attending mental health clinics in Tanzania.<br>The objectives are well-defined, and the inclusion criteria for the study are well-described. The authors present a good flowchart of the recruitment of people for the study, respecting ethical principles.<br>The subject of the paper is very relevant due to the complexity of the diagnostics of cysticercosis and the relevance of the disease, that can lead to epilepsy.<br>As I mentioned, the article is well written and can be easier to read and understand, with some improvements in some confusing parts, especially in the results section.	We thank the reviewer for the complement
**Reviewer #3:** In Taenia solium endemic communities with limited hygienic infrastructure, epilepsy-associated neurocysticercosis become a major public health problem that requires improvement in diagnostic capacities and intervention strategies. People living in contact with subjects infected with Taenia Solium are at higher risk of acquiring parasite eggs through the fecal-oral route that eventually become larvae in several tissues including the central nervous system. While larvae become calcified in the tissues, several neurological complications are produced, including epilepsy. Neuroimaging is the most reliable diagnostic tool, but access is limited by high cost where it is more needed. Alternatively, several efforts have been done to develop sensitive and specific serological tools (ELISA and Westernblot) to increase diagnostic capacities in endemic areas. The manuscript by Charles E. Makasia and coworkers is following the serological approach and described the correlation between a serological test for neurocysticercosis and neuroimaging studies.<br>L144: PWE is already defined “L 116” no need	

to repeat.<br>L159: “consecutively selected” please replace with “enrolled prospectively”<br>L160: consider “with or without” … anti-seizure medication<br>L169 – 190: Consider shortening, too much detail. Epilepsia definition should be included in the inclusion criteria. Avoid text repetition.<br><br>These reviewers consider this is an important contribution, but extensive description dilutes the main findings.	**Done:** L144: PWE already defined in “L 116” “People with epilepsy” deleted to avoid repetition.<br>Done: L159: “consecutively selected” replaced with “enrolled prospectively”<br>**Done:** L160: “with or without” … anti-seizure medication considered instead of “on and off”
**Post re-submission comments**<br><br><br>Some material included in your submission may be copyrighted. According to PLOSu2019s copyright policy, authors who use figures or other material (e.g., graphics, clipart, maps) from another author or copyright holder must demonstrate or obtain permission to publish this material under the Creative Commons Attribution 4.0 International (CC BY 4.0) License used by PLOS journals. Please closely review the details of PLOSu2019s copyright requirements here: PLOS Licenses and Copyright. If you need to request permissions from a copyright holder, you may use PLOS's Copyright Content Permission form.<br>Please respond directly to this email and provide any known details concerning your material's license terms and permissions required for reuse, even if you have not yet obtained copyright permissions or are unsure of your material's copyright compatibility. Once you have responded and addressed all other outstanding technical requirements, you may resubmit your manuscript within Editorial Manager.<br>Potential Copyright Issues	All recommended items have been taken care of as suggested but one more special to us was the issue of site maps which has been dealt with as follows;<br><br>We appreciate and agree with this advice, however, sticking to the copyright policy became a little bit challenging since some of the owners of the materials were not responding to our requests. To avoid this, we decided to develop our materials, therefore we generated the site maps by using QGIS software version 3.34.2. However, our maps were substandard and therefore, we decided that we were going to use texts only and avoid maps to describe the study areas.

NB. Line numbers have changed due to the addition of recommended parts and the re-arrangement of the document.

A track-changed version has been prepared and can be attached to this resubmission.

The following items; Declarations **of interest, Ethics Statement, Data statement, Acknowledgment, Funding, and Author Contributions** have been removed from the supporting information file as recommended.

A clean final copy has also been prepared; where possible, retrimmed to accommodate the required shortened sections as recommended, but precautions have been taken to avoid diverging the original meaning of sentences.

Yours sincerely

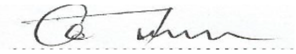

Charles E. Makasi

Corresponding Author
