# Supplementary material for: Correlation of serum screening and neuroimaging studies: A comparative analysis for diagnostic evaluation of neurocysticercosis among people with epilepsy attending mental health clinics in selected district hospitals of Tanzania
Source: PLoS Negl Trop Dis. 2025 Apr 10;19(4):e0013002. doi: 10.1371/journal.pntd.0013002 (PMC12040232; doi:10.1371/journal.pntd.0013002)
Supplement: Attachment — Submitted filename: Response to Reviewers.pdf [file pntd.0013002.s003.pdf]

Manuscript Number: PNTD-D-24-01083R1

Title: Correlation of serum screening and neuroimaging studies: a comparative analysis for diagnostic evaluation of neurocysticercosis among people with epilepsy attending mental health clinics in selected district hospitals of Tanzania

Authors: Charles E. Makasi, Bernard Ngowi, Andrew Kilale, Godfrey Guga, Michael J. Mahande, Johnson Mshiu, Abisai Kisinda, Blandina T. Mmbaga.

Journal: PLOS Neglected Tropical Diseases

Subject: Author's response to reviewer's comments

Dear Editor,

Thanks very much for the invitation to resubmit our manuscript entitled: **"Correlation of serum screening and neuroimaging studies: a comparative analysis for diagnostic evaluation of neurocysticercosis among people with epilepsy attending mental health clinics in selected district hospitals of Tanzania.".** We appreciate the reviewers' useful comments and suggestions to improve our manuscript. The following were comments from the reviewers on our manuscript;

Methods

-Are the objectives of the study clearly articulated with a clear testable hypothesis stated?

-Is the study design appropriate to address the stated objectives?

-Is the population clearly described and appropriate for the hypothesis being tested?

-Is the sample size sufficient to ensure adequate power to address the hypothesis being tested?

-Were correct statistical analysis used to support conclusions?

-Are there concerns about ethical or regulatory requirements being met?

Reviewer #1: The revised format has incorporated all suggestions. However, the objectives mentioned by the author as a reply could be written after mentioning the aim of the study at the end of Introduction section of the article. Rest all looks good to go. All the best or final proofreading and publication.

Reviewer #2: The suggested modifications have been made, upgrading the paper. The methodology is now more clear.

Reviewer #4: -Are the objectives of the study clearly articulated with a clear testable hypothesis stated? YES
-Is the study design appropriate to address the stated objectives? YES
-Is the population clearly described and appropriate for the hypothesis being tested? YES
-Is the sample size sufficient to ensure adequate power to address the hypothesis being tested? NO
-Were correct statistical analysis used to support conclusions? YES
-Are there concerns about ethical or regulatory requirements being met? NO

Results

-Does the analysis presented match the analysis plan?

-Are the results clearly and completely presented?

-Are the figures (Tables, Images) of sufficient quality for clarity?

Reviewer #1: Yes, the suggested corrections have been done by the author.

Reviewer #2: The suggested modifications have been made. The results are more clear and easier to understand.

Reviewer #4: -Does the analysis presented match the analysis plan? YES
-Are the results clearly and completely presented? YES
-Are the figures (Tables, Images) of sufficient quality for clarity? NO

Conclusions

-Are the conclusions supported by the data presented?

-Are the limitations of analysis clearly described?

-Do the authors discuss how these data can be helpful to advance our understanding of the topic under study?

-Is public health relevance addressed?

Reviewer #1: Yes. The author has mentioned conclusion appropriately along with addressing the public health relevance in the revised document.

Reviewer #2: The conclusions were improved as was suggested by the reviewers, and are now aligned with the objectives of the work

Reviewer #4: -Are the conclusions supported by the data presented? NO
-Are the limitations of analysis clearly described? YES
-Do the authors discuss how these data can be helpful to advance our understanding of the topic under study? YES
-Is public health relevance addressed? YES

Editorial and Data Presentation Modifications?

Use this section for editorial suggestions as well as relatively minor modifications of existing data that would enhance clarity. If the only modifications needed are minor and/or editorial, you may wish to recommend "Minor Revision" or "Accept".

Reviewer #1: Minor revision: Adding the objectives of the study at the end of introduction in the main article file

Reviewer #2: Despite the corrections made, there are some small changes that need to be made
lines210, 211 - Should be "ml", instead of "mls"
Lines 217, 232 - should be "ºC", instead of "degree centigrade"
Line 318 - should be "p=0.030", not p<0.03 (according the table 3)
Line 320 - should be "p=0.024", not p<0.024 (according the table 3)
Lines 553, 557,571,575,586,609,617,627,631,648,652,657,665 - You missed the italics

Reviewer #4: The authors have done great work in assessing the "Correlation of serum screening and neuroimaging studies: a comparative analysis for diagnostic evaluation of neurocysticercosis among people with epilepsy attending mental health clinics in selected district hospitals of Tanzania"
The manuscript has the ability to contribute to the scientific body of knowledge regarding decisions making on who should be taken for neuroimaging in T. solium endemic areas. However, the manuscript has a number of typos and areas requiring clarifications. Please refer to the following comments and suggestions

INTRODUCTION
Line 102: Kindly replace the word "Guts" with intestines as guts is more colloquial
Line 104: The placing of porcine cysticercosis in brackets at the end of the sentence is misleading as it now means that T. solium larval stage is called porcine cysticercosis.
Line 113: The phrase T. solium-caused conditions should be replaced with T. solium associated conditions. This is because T. solium per say does not cause epilepsy.
Line 121-123: The techniques for diagnosis of NCC are available (as explained in line 126) as they have been published. However, its the availability, knowledge and lack of equipment to perform the tests that is challenging. The authors should consider rephrasing the sentence and combine with the follow up statement in line 124-126.
Line 134: Remove the words "But also" and start as a new paragraph.

METHODS
Line 144: Indicate early here when the study was conducted and the dates can then be removed from line 165 and don't need to be mentioned again in line 177.
Line 146: Remove the word respectively and indicate the regions for the two study sites e.g Kongwa district hospital in Dodoma, central Tanzania and Chunya hospital in Mbeya, Southern Tanzania.
Line 149: Only mention Makasi et., al. once and enter the citations e.g. as described by Makasi et al (21,22)
Line 169: The authors should define the abbreviation "CT" and thereafter use the abbreviation CT scan
Line 176: The authors do not need to state that CM was an employee of the study. Also check the sentence "attended potential participants" and instead write "attended to potential participants"

PROCEDURES:
Overall, this paragraph needs to be restructured. There is no need to mention each district hospital separately with the specific days when the mental health clinics are held (unless this has an effect on the results). In addition, it has already been mentioned in the study design and setting that the study was done in Kongwa and Chunya district hospitals. The authors should focus on outlining the procedures done during the study such as described from line 186. I find the included information from line 181 to part of 186 unnecessary for the procedures section.

Line 194: What do the authors mean by "convenience was applied"?
Line 198: Add "s" to participant. Also replace "whereby" with "and" specify if the written information is written informed consent signed
Line 200: The authors refer to collection of biological samples. What are these other biological samples collected in addition to stool and blood samples? In any case this section is about data collection methods and tools therefore, collection of samples should move downwards to Test methods
Line 201: Rephrase the sentence to read "After sample collection, further data was collected from participants by CM and SN, the trained medical practitioners with neurological experience."
The mentioning of DS (which does not appear anywhere else in the manuscript) and CYSTINET-Africa is not necessary here.
Line 207: What do the authors mean by "and neurological status by the use of in-depth neurological and neurological examination standardized and validated forms"? This statement is confusing
Line 212-214: This statement indicates that the Red top bottles were left at room temperature for one hour and serum was obtained. However, the subsequent sentence (line 214) indicates that the Red top containers were left in the fridge and serum only obtained after centrifugation. The authors should phrase these sentences correctly to avoid confusion of the process.

Line 216: The table may be removed if the authors just state that serum was collected in three cryovials. One for testing with apDia Ag-ELISA, the second for LDBio IgG Western blot and the third for quality control.
Line 217: kindly complete the sentence "similar barcodes/unique…..?"
Line 229: Change the phrase "certain amount of sample"… to "certain number of samples" If the

agreement was to send samples when they were 25 or more, the authors should state so without creating ambiguity like a certain number of samples.
Line 230-232: Kindly include appropriate punctuations to make the sentence easy to understand. If the professional laboratory experts are the trained laboratory staff then state as such
Line 234: Replace the sentence “with an escort of the laboratory expert” with “accompanied by a trained laboratory staff”
Line 236: replace “done” with “performed” and “procedures” with “guidelines”
Line 241: Replace “every” with “all” and “for one” with “on either”
Line 242-243: state that the CT scan examination was performed as described by Makasi et al 2024. and no need to highlight it in board colour
Line 243: Remove “The” from the beginning of the sentence.
Line 244: Remove “The” before NCC
Line 247: The authors to explain what CR represents. In addition, the authors use so many abbreviations for names of persons and these abbreviations are not stated anywhere. The authors should just state what was done without the trouble of indicating exactly the names of people who performed certain functions.
Line 249: The authors have already stated that the revised 2017 Del Brutto criteria was used to make NCC diagnosis. This is a repetition.
Line 251: What does (source) represent?
Line 252-253: The authors should simply state that treatment options for patients with NCC was agreed upon by the study clinical team which also included a neurologist. The abbreviated names will appear in the acknowledgments.
Line 256: The authors should state what treatment was given to the patients with NCC
Line 283: The abbreviations Ab, Ag have not been defined anywhere before

RESULTS
Line 283: Delete the sentence “i.e kongwa and Chunya”
Line 286: The authors should begin by presenting first the demographic data before presenting the results of the serological tests and CT scan examination.
Line 286-287: the sentence “neuroimaging (cerebral computed tomography (CT) scan examination)” should read (cerebral CT scanning). Provided the CT abbreviation is explained as suggested for line 169 above
Fig 1: A box is missing to explain those CC-ve without neurological signs/symptoms (somewhere between N=196 and N=23)
304-305: Replace “pattern in” with “pattern in terms of”.. and place a full stop instead of a coma after participants.
305: Replace the sentence “more than twice PWE” to “more than twice the number of PWE”
306: Replace the sentence “twice as many as farmers” to “twice the proportion of farmers”
Line 317 and Table 3 cysticercosis positive: The authors should indicate that the 25 CC+ were positive on either test.
Line 320: delete the repetition of “i.e Kongwa; 13. (7.6%) vs Chunya 4 (7.8%) This already mentioned above.
330-331: The authors should check the phrasing of the sentence especially the last part of the sentence.
Table 4: The results of the table can be incorporated into Table 3 under Neurocysticercosis. This way the 7 and 1 CC positive participants would be known in terms of the hospital they came

from.
Table 5: This is not necessary as it is simply explained in the text
Line 350; check the value for the PPV

DISCUSSION
Line 390: Consider replacing the sentence “was very dangerous.” The authors may consider using “had its own complications”
Line 404: remove “…for infection” after the specificity in the brackets

We were able to address all the comments and suggestions, and therefore we are expecting that the paper can now be accepted in your journal. Please find our point-by-point responses to the reviewers’ comments and concerns in the tables below:

Table 1. Response to Reviewers’ comments (Comments to the Author)

**Comment**	**Response**
**Methods**	
Reviewer #1: The revised format has incorporated all suggestions. However, the objectives mentioned by the author as a reply could be written after mentioning the aim of the study at the end of the Introduction section of the article. Rest all looks good to go. All the best or final proofreading and publication.	
Reviewer #2: The suggested modifications have been made, upgrading the paper. The methodology is now more clear..	I would like to express my appreciation for recognizing the correction that has been made Thank you for your positive and encouraging feedback.
Reviewer #4: -Are the objectives of the study clearly articulated with a clear testable hypothesis stated? YES<br>-Is the study design appropriate to address the stated objectives? YES<br>-Is the population clearly described and appropriate for the hypothesis being tested? YES<br>-Is the sample size sufficient to ensure adequate power to address the hypothesis being tested? NO<br>-Were correct statistical analysis used to support conclusions? YES	Thank you for your positive feedback on the objective, study design, population description, statistical analysis, and free from concerns about ethical issues.<br>Otherwise, we declared the insufficiency of the sample size in the manuscript at section 7.2 Limitation. Recognizing the importance of adequate power to address the hypothesis, we appropriate statistical methods to account for the issue and robustness of the findings

-Are there concerns about ethical or regulatory requirements being met? NO	
**Results**	
-Does the analysis presented match the analysis plan?<br>-Are the results clearly and completely presented?<br>-Are the figures (Tables, Images) of sufficient quality for clarity?	
Reviewer #1: Yes, the suggested corrections have been done by the author.	Reviewer #1: I appreciate your positive feedback and confirmation that the correction has been taken care.
Reviewer #2: The suggested modifications have been made. The results are more clear and easier to understand.	Reviewer #2: Thank you for your invaluable suggestions and we are happy to hear that you are satisfied with the revision that you have recommended.
Reviewer #4: -Does the analysis presented match the analysis plan? YES<br><br>-Are the results clearly and completely presented? YES<br><br>-Are the figures (Tables, Images) of sufficient quality for clarity? NO	Thank you for your thoughtful comments. We acknowledge your appreciation for the matching of the analysis and analysis plan as well as the clarity and completeness of the results.<br>I understand the importance of the figures (Tables and images). We have revised and tried our level best to meet the accepted appropriate quality.
**Conclusion**	
-Are the conclusions supported by the data presented?<br>-Are the limitations of analysis clearly described?<br>-Do the authors discuss how these data can be helpful to advance our understanding of the topic under study?<br>-Is public health relevance addressed?	
Reviewer #1: Yes. The author has mentioned conclusion appropriately along with addressing the public health relevance in the revised document	We thank reviewer #1 for the supportive comment which has contributed to the improvement of our manuscript.
Reviewer #2: The conclusions were improved as was suggested by the reviewers, and are now aligned with the objectives of the work	Reviewer #2 is highly appreciated for recognizing our task as per recommended instructions, but the improvement obtained has

	been achieved through this reviewer's constructive comment.
Reviewer #4: -Are the conclusions supported by the data presented? NO<br>-Are the limitations of analysis clearly described? YES<br>-Do the authors discuss how these data can be helpful to advance our understanding of the topic under study? YES<br>-Is public health relevance addressed? YES	We thank reviewer #4 for the thoughtful insight on our manuscript, however, we recognized this reviewer's concern about the conclusion part not being supported by the data presented. We revised our results and worked on the conclusion again to make sure that they aligned.

……..…. END OF REVIEWER S' COMMENT(S) AND RESPONSES………...

Table 2. Editorial and Data Presentation Modifications

Comment	Response
Reviewer #1: Minor revision: Adding the objectives of the study at the end of introduction in the main article file	Thank you for your insights, the objective of this study was stated in the preceding sentence before the last one in the introduction part which explains the STARD Format. However, sticking to your advice we have rephrased the last sentence in the introduction part.
Reviewer #2: Despite the corrections made, there are some small changes that need to be made<br>lines210, 211 - Should be "ml", instead of "mls"<br>Lines 217, 232 - should be "ºC", instead of "degree centigrade"<br>Line 318 - should be "p=0.030", not p<0.03 (according the table 3)<br>Line 320 - should be "p=0.024", not p<0.024 (according the table 3)<br>Lines 553, 557,571,575,586,609,617,627,631,648,652,657,665 - You missed the italics	**Done**: lines210, 211 - Should be "ml", instead of "mls"<br>**Done:** Lines 217, 232 - should be "ºC", instead of "degree centigrade"<br>**Done:** Line 318 - should be "p=0.030", not p<0.03 (according the table 3)<br>**Done:** Line 320 - should be "p=0.024", not p<0.024 (according the table 3)<br><br>**Done:** Lines 553, 557,571,575,586,609,617,627,631,648,652,657,665 - You missed the italics

Reviewer #4: The authors have done great work in assessing the "Correlation of serum screening and neuroimaging studies: a comparative analysis for diagnostic evaluation of neurocysticercosis among people with epilepsy attending mental health clinics in selected district hospitals of Tanzania" The manuscript has the ability to contribute to the scientific body of knowledge regarding decisions making on who should be taken for neuroimaging in T. solium endemic areas. However, the manuscript has several typos and areas requiring clarifications. Please refer to the following comments and suggestions.	
INTRODUCTION<br>Line 102: Kindly replace the word "Guts" with intestines as guts is more colloquial<br>Line 104: The placing of porcine cysticercosis in brackets at the end of the sentence is misleading as it now means that T. solium larval stage is called porcine cysticercosis.<br>Line 113: The phrase T. solium-caused conditions should be replaced with T. solium associated conditions. This is because T. solium per say does not cause epilepsy.<br>Line 121-123: The techniques for diagnosis of NCC are available (as explained in line 126) as they have been published. However, its the availability, knowledge and lack of equipment to perform the tests that is challenging. The authors should consider rephrasing the sentence and combine with the follow up statement in line 124-126.<br>Line 134: Remove the words "But also" and start as a new paragraph.	**Done:** Line 102: Kindly replace the word "Guts" with intestines as guts is more colloquial<br>**Done and the sentence has been rephrased:** The placing of porcine cysticercosis in brackets at the end of the sentence is misleading as it now means that T. solium larval stage is called porcine cysticercosis<br>**Done:** Line 113: The phrase T. solium-caused conditions should be replaced with T. solium associated conditions. This is because T. solium per say does not cause epilepsy.<br><br>**Line 121-123 rephrased**<br><br>**Line 134 rephrased**
METHODS<br>Line 144: Indicate early here when the study was conducted and the dates can then be removed from line 165 and don't need to be mentioned again in line 177.	**Done, dates of study conduct remained one only, other two have been deleted.**

Line 146: Remove the word respectively and indicate the regions for the two study sites e.g Kongwa district hospital in Dodoma, central Tanzania and Chunya hospital in Mbeya, Southern Tanzania.<br>Line 149: Only mention Makasi et., al. once and enter the citations e.g. as described by Makasi et al (21,22)<br>Line 169: The authors should define the abbreviation "CT" and thereafter use the abbreviation CT scan<br>Line 176: The authors do not need to state that CM was an employee of the study. Also check the sentence "attended potential participants" and instead write "attended to potential participants"	Line 146; Done, removed the word "respectively" as recommended.<br>Line 149, only one Makasi et al left as instructed.<br>Done; erased the word employee of the study; "attended potential participants" replaced by "attended to potential participants"
PROCEDURES:<br>Overall, this paragraph needs to be restructured. There is no need to mention each district hospital separately with the specific days when the mental health clinics are held (unless this has an effect on the results). In addition, it has already been mentioned in the study design and setting that the study was done in Kongwa and Chunya district hospitals. The authors should focus on outlining the procedures done during the study such as described from line 186. I find the included information from line 181 to part of 186 unnecessary for the procedures section.<br>Line 194: What do the authors mean by "convenience was applied"?<br>Line 198: Add "s" to participant. Also replace "whereby" with "and" specify if the written information is written informed consent signed<br>Line 200: The authors refer to collection of biological samples. What are these other biological samples collected in addition to stool and blood samples? In any case this section is about data collection methods and tools therefore, collection of samples should move downwards to Test methods<br>Line 201: Rephrase the sentence to read "After sample collection, further data was collected from participants by CM and SN, the trained medical practitioners with neurological experience."<br>The mentioning of DS (which does not appear anywhere else in the manuscript) and CYSTINET-	Done: Days of clinic have been removed<br>Line 194 means A convenience sampling technique. The sentence has been rephrased and reads "A convenience sampling technique was applied during recruitment, and other attendees to the mental clinic with problems other than epilepsy were not eligible for the study"<br>Line 198 Done as suggested<br>Line 200: thank you for your observation, we have removed description of sample collection from the Data collection and tools section and remained with what has been described in the test methods section.<br>**Line 201** rephrased and unnecessary terms removed

Africa is not necessary here.<br>Line 207: What do the authors mean by “and neurological status by the use of in-depth neurological and neurological examination standardized and validated forms”? This statement is confusing<br>Line 212-214: This statement indicates that the Red top bottles were left at room temperature for one hour and serum was obtained. However, the subsequent sentence (line 214) indicates that the Red top containers were left in the fridge and serum only obtained after centrifugation. The authors should phrase these sentences correctly to avoid confusion of the process.<br>Line 216: The table may be removed if the authors just state that serum was collected in three cryovials. One for testing with apDia Ag-ELISA, the second for LDBio IgG Western blot and the third for quality control.<br>Line 217: kindly complete the sentence “similar barcodes/unique…..?”<br>Line 229: Change the phrase “certain amount of sample”… to “certain number of samples” If the agreement was to send samples when they were 25 or more, the authors should state so without creating ambiguity like a certain number of samples.<br>Line 230-232: Kindly include appropriate punctuations to make the sentence easy to understand. If the professional laboratory experts are the trained laboratory staff then state as such<br>Line 234: Replace the sentence “with an escort of the laboratory expert” with “accompanied by a trained laboratory staff”<br>Line 236: replace “done” with “performed” and “procedures” with “guidelines”<br>Line 241: Replace “every” with “all” and “for one” with “on either”<br>Line 242-243: state that the CT scan examination was performed as described by Makasi et al 2024. and no need to highlight it in board colour<br>Line 243: Remove “The” from the beginning of the sentence.<br>Line 244: Remove “The” before NCC<br>Line 247: The authors to explain what CR represents. In addition, the authors use so many	 Thank you for pointing out a confusion in our statement; we meant that; “The neurological status was assessed through a detailed neurological examination, using standardized and validated forms to ensure consistency and accuracy in data collection” We paraphrased the statement to avoid confusion<br>Line 212-214; we have removed the second description of the serum harvest through centrifugation<br><br>Line 216; Table removed and the following statement was inserted as suggested “The serum was collected in three cryovials; one for testing with apDia Ag-ELISA, the second for LDBio IgG Western blot, and the third for quality control”. The rest of tables have been re-numbered.<br><br>Thank you very much for the reminder; the sentence has been completed and it reads “Similar barcodes/unique were attached to the tubes, showing that all three aliquots belonged to one individual”<br>Line 229: sentence rephrased to state exactly 25 sample or more.<br><br>Line 230-232 proper punctuation and rephrasing done. The new sentence reads” Frozen samples were well labeled and prepared by trained laboratory staff, adhering to all biohazard sample regulations. They were then packed into liquid nitrogen tanks, which maintained the shipping temperature at -20 ºC or below”<br>Line 234 “with an escort of the laboratory expert” replaced with “accompanied by a trained laboratory staff”<br>Done: Line 236: replace “done” with “performed” and “procedures” with “guidelines”<br>Done: Line 241: Replace “every” with “all” and “for one” with “on either”<br>**Done:** Line 242-243: state that the CT scan examination was performed as described by Makasi et al 2024. and no need to highlight it in the board colour<br>**Done:** Line 243: Remove “The” from the beginning of the sentence.

abbreviations for names of persons and these abbreviations are not stated anywhere. The authors should just state what was done without the trouble of indicating exactly the names of people who performed certain functions.<br>Line 249: The authors have already stated that the revised 2017 Del Brutto criteria was used to make NCC diagnosis. This is a repetition.<br>Line 251: What does (source) represent?<br>Line 252-253: The authors should simply state that treatment options for patients with NCC was agreed upon by the study clinical team which also included a neurologist. The abbreviated names will appear in the acknowledgments.<br>Line 256: The authors should state what treatment was given to the patients with NCC<br>Line 283: The abbreviations Ab, Ag have not been defined anywhere before	**Line 244:** "The" was removed before NCC, but a long term of NCC has been replaced to avoid starting a sentence with an abbreviation.<br>Line 247: All abbreviated names have been removed as suggested.<br>**Line 249:** the sentence with repetition of revised 2017 Del Brutto criteria has been removed.<br>Line 251: What does (source) represent? It was an unaddressed query from supervisors asking for a reference, it has been replaced by a proper citation.<br>Done: Line 252-253: The authors should simply state that treatment options for patients with NCC were agreed upon by the study clinical team which also included a neurologist.<br>Line 256: Antiparasitic (a combined albendazole and praziquantel) treatment.<br>**Done:** Line 283: The abbreviations Ab, Ag have not been defined anywhere before
RESULTS<br>Line 283: Delete the sentence "i.e kongwa and Chunya"<br>Line 286: The authors should begin by presenting first the demographic data before presenting the results of the serological tests and CT scan examination.<br>Line 286-287: the sentence "neuroimaging (cerebral computed tomography (CT) scan examination)" should read (cerebral CT scanning). Provided the CT abbreviation is explained as suggested for line 169 above<br>Fig 1: A box is missing to explain those CC-ve without neurological signs/symptoms (somewhere between N=196 and N=23)<br>304-305: Replace "pattern in" with "pattern in terms of".. and place a full stop instead of a coma after participants.<br>305: Replace the sentence "more than twice PWE" to "more than twice the number of PWE"<br>306: Replace the sentence "twice as many as farmers" to "twice the proportion of farmers"<br>Line 317 and Table 3 cysticercosis positive: The authors should indicate that the 25 CC+ were positive on either test.<br>Line 320: delete the repetition of "i.e Kongwa; 13. (7.6%) vs Chunya 4 (7.8%) This already mentioned above.	**Done:** Line 283: Delete the sentence "i.e kongwa and Chunya"<br><br>Line 286: We appreciate the observation which has been addressed by the reviewer. The demographic data was provided first in this manuscript, backed up by a flowchart, and the CT scan information in the next paragraph is an extension of that information. We have reviewed the section and connected it to form a single, coherent paragraph that connects the demographic data to the serological and CT findings as shown in the flowchart in order to improve clarity and ensure that the logical flow makes sense.<br>**Done:** Line 286-287: the sentence "neuroimaging (cerebral computed tomography (CT) scan examination)" should read (cerebral CT scanning). Provided the CT abbreviation is explained as suggested for line 169 above<br>Figure 1. A box has been inserted explaining Cysticercosis negative without neurological signs/symptoms N=173 as recommended.<br>Done: 304-305: Replace "pattern in" with "pattern in terms of".. and place a full stop instead of a coma after participants.<br>Done: 306: Replace the sentence "twice as many as farmers" to "twice the proportion of farmers"

330-331: The authors should check the phrasing of the sentence especially the last part of the sentence.<br>Table 4: The results of the table can be incorporated into Table 3 under Neurocysticercosis. This way the 7 and 1 CC positive participants would be known in terms of the hospital they came from.<br>Table 5: This is not necessary as it is simply explained in the text<br>Line 350; check the value for the PPV	Done: Line 317 and Table 3 cysticercosis positive: The authors should indicate that the 25 CC+ were positive on either test.<br>**Done:** Line 320: delete the repetition of "i.e Kongwa; 13. (7.6%) vs Chunya 4 (7.8%) This already mentioned above.<br>**Table 4:** We would like to thank the reviewer for this observation and for the advice on combining the two tables. However, these tables show two different items. Table 3 (which is now re-numbered Table 2); Distribution of serum cysticercosis tests and neuroimaging (Computed Tomography); provides a broad overview of the distribution of cysticercosis tests and CT scans across research participants, and therefore, is more of the descriptive table. On the other hand, Table 4 (Re-numbered Table 3). Serum cysticercosis test results vs computed tomography for neurocysticercosis (N=36); is analytic, aiming to display the diagnostic accuracy of the serum cysticercosis test in detecting neurocysticercosis in comparison to CT scanning. Therefore, we think that combining them might bring some confusion to some of the readers but we stand to be corrected.<br>Done, table deleted: Table 5: This is not necessary as it is simply explained in the text<br>Line 350; check the value for the PPV: thank you for your comment, but unfortunately, I am not very clear with the comment. Otherwise, it matched the PPV displayed in the table which is 38.9.0% which is still more than a quarter
DISCUSSION<br>Line 390: Consider replacing the sentence "was very dangerous." The authors may consider using "had its own complications"<br>Line 404: remove "…for infection" after the specificity in the brackets	**Done:** Line 404: remove "…for infection" after the specificity in the brackets

END OF EDITORIAL AND DATA PRESENTATION MODIFICATIONS AND THEIR RESPONSES

NB. Line numbers have changed due to the addition of recommended parts and the re-arrangement of the document.
A track-changed version has been prepared and can be attached to this resubmission.
A clean final copy has also been prepared; where possible, retrimmed to accommodate the required shortened sections as recommended, but precautions have been taken to avoid diverging the original meaning of sentences.

Yours sincerely

Charles E. Makasi
