# Supplementary material for: Correlation of serum screening and neuroimaging studies: A comparative analysis for diagnostic evaluation of neurocysticercosis among people with epilepsy attending mental health clinics in selected district hospitals of Tanzania
Source: PLoS Negl Trop Dis. 2025 Apr 10;19(4):e0013002. doi: 10.1371/journal.pntd.0013002 (PMC12040232; doi:10.1371/journal.pntd.0013002)
Supplement: Attachment — Submitted filename: Response to Reviewers.docx [file pntd.0013002.s004.docx]

Dr. Charles E. Makasi

22^nd^ February 2025

Dear

Feng Xue

Guest Editor

PLOS Neglected Tropical Diseases

**Re: Rebuttal letter on point-by-point response to reviewers’ comments**

Dear Feng Xue,

Thank you for considering publishing our manuscript PNTD-D-24-01083R2 titled;

**“Correlation of serum screening and neuroimaging studies: a comparative analysis for diagnostic evaluation of neurocysticercosis among people with epilepsy attending mental health clinics in selected district hospitals of Tanzania”.**

I am glad to submit our response to the comments from the reviewers on the PLOS Neglected Tropical Diseases as required.

This submission includes the following items;

- A rebuttal letter responding to each point the editor and reviewer(s) raised.
- A copy of the revised manuscript with Track Changes.
- A clean copy of the revised paper without tracked changes.

**POINT-BY-POINT RESPONSE TO REVIEWERS' COMMENTS**

**METHODS**:

| **Reviewer** | **Journal requirement** | **Response** |
| --- | --- | --- |
|  | **Are the study objectives clearly articulated with a clear testable hypothesis stated?** | We highly appreciate the positive comments on all aspects of the methods section. |
| **Reviewer #1:** | The revised version is clear & concise with all suggested modifications. |  |
| **Reviewer #2:** | Yes |  |
| **Reviewer #4:** | Yes |  |
|  | **Is the study design appropriate for addressing the stated objectives?** |  |
| **Reviewer #2:** | Yes |  |
| **Reviewer #4:** | Yes |  |
|  | **Is the population clearly described and appropriate for the hypothesis being tested?** |  |
| **Reviewer #2:** | Yes |  |
| **Reviewer #4:** | Yes |  |
|  | **Is the sample size sufficient to ensure adequate power to address the hypothesis being tested?** |  |
| **Reviewer #2:** | Yes |  |
| **Reviewer #4:** | Yes |  |
|  | **Were correct statistical analyses used to support conclusions?** |  |
| **Reviewer #4:** | - YES |  |
|  | **Are there concerns about ethical or regulatory requirements being met?** |  |
| **Reviewer #2:** | NO |  |
| **Reviewer #4:** | NO |  |

**RESULTS**:

| **Reviewer** | **Journal requirement** | **Response** |
| --- | --- | --- |
|  | **Does the analysis presented match the analysis plan?** | We thank the reviewers for their compliments on the results section as it is the main part of the manuscript. |
| **Reviewer #1:** | Yes, the results are complete with a clear presentation. |  |
| **Reviewer #2:** | Yes |  |
| **Reviewer #4:** | Yes |  |
|  | **Are the results clearly and completely presented?** |  |
| **Reviewer #2:** | Yes |  |
| **Reviewer #4:** | Yes |  |
|  | **Are the figures (Tables, Images) of sufficient quality for clarity?** |  |
| **Reviewer #2:** | Yes |  |
| **Reviewer #4:** | Yes |  |

**CONCLUSIONS**:

| **Reviewer** | **Journal requirement** | **Response** |
| --- | --- | --- |
|  | **Are the conclusions supported by the data presented?** | As authors, we are grateful that the reviewers have given positive comments on the drawn conclusions for the study. |
| **Reviewer #1:** | Conclusion along with strengths and limitations described appropriately. Public health relevance has been addressed as well. |  |
| **Reviewer #2:** | Yes |  |
| **Reviewer #4:** |  |  |
|  | **Are the limitations of analysis clearly described?** |  |
| **Reviewer #2:** | Yes |  |
| **Reviewer #4:** | Yes |  |
|  | **Do the authors discuss how these data can be helpful to advance our understanding of the topic under study?** |  |
| **Reviewer #2:** | Yes |  |
| **Reviewer #4:** | No |  |
|  | **Is public health relevance addressed?** |  |
| **Reviewer #2:** | Yes |  |
| **Reviewer #4:** | Yes |  |

**GENERAL COMMENTS**

| **Reviewer** | **Comment** | **Response** |
| --- | --- | --- |
| **Reviewer #4:** | 1. The study aims to determine the correlation between serum screening and neuroimaging studies to diagnose neurocysticercosis. However, the authors have not described the NCC lesions seen from the neuroimaging examination for an informed decision regarding correlation with serum screening. This description would enhance our understanding of the correlation | To respond to this, the manuscript includes a table of the distribution, number of lesions, location, and stages of cysts with its guiding description. |
|  | 2. Line 310: The authors stated in the methods that diagnosis of NCC would be based on the revised Del Brutto 2017 criteria and would define probable and definite NCC. However, there is no mention anywhere in the results about this but here they refer to a Gold Standard test without mentioning the Del Brutto 2017 Criteria. The authors can shed more light on this. | Thank you very much for your constructive observation. We meant that Neuroimaging is considered the gold standard method for the detection of NCC; however, it is highly dependent on the Del Brutto criteria as a valuable diagnostic tool. We have revised the statement to show what we meant. |
|  | 3. Line 402-414: The authors have not discussed sensitivity and specificity in the context of their findings. For example, the cited paper of Santini et al. discussed their findings. It would greatly improve our understanding of the two parameters discussed within the context of the authors findings/results. The same applies to the likelihood ratio. The authors have at least made reference to their findings in the discussion of PPV/NPV and ROC | We have reviewed the text and separated between the cited *Santini et al*., study and the results from our study.  Therefore, we revised the sensitivity and specificity part, we also re-wrote the writing at parts of Predictive values, and ROC sections to bring more sense. |
|  | 1. Line 475-488: The authors correctly state the multiple analysis used as a strength of their study. However, the fact that participants were tested with cysticercosis tests and that neuroimaging was performed cannot be considered as strengths unless they state that all CC positives had Neuroimaging to be used for their comparative analysis. An explanation for this would help understand their reasoning. | Thank you for your comment; we have paraphrased the strength section to align with what has been recommended. |

**EDITORIAL AND DATA PRESENTATION MODIFICATIONS?**

| **Reviewer** | **Comment** | **Response** |
| --- | --- | --- |
| **Reviewer #1:** | Nil |  |
| **Reviewer #2:** | Despite the corrections made, some small changes still need to be made | The changes have been made by italicizing the words as required |
|  | Lines 656, and 673 - missed the italics (Leishmania; Helicobacter pylori) |  |
| **Reviewer #4:** | Minor |  |
|  | Line 165: The authors should define here the abbreviation "CT" e.g. Computed tomography (CT) scans. After this, the authors can now just use "CT scan" without writing it in full. | The abbreviation “CT scan” has been defined as required. Subsequent sections have used the abbreviation, accordingly. |
|  | Line 269: As suggested earlier the (cerebral computed tomography (CT) scan examination) can be written simply as (cerebral CT scan examination) if the CT abbreviation is defined earlier as suggested above. | Done as suggested |
|  | Line 288: Consider replacing the sentence ".....more than twice PWE without..." with "more than twice the number of PWE without..." | Thank you. This has been done. |
|  | Line 312-312: The authors should check this sentence. Add % to (87.5). What does the last part mean (...and 1 (12.5%) case which could not be picked serum cysticercosis test)??? | Thank you. This has been done and the last part has been elaborated. |
|  | Line 328: the PPV is written as 38.9.0% is this the correct nomenclature or did the authors mean 38.9%? | Thank you for seeing the typo, now 38.9.0% reads 38.9%. |

**SUMMARY AND GENERAL COMMENTS**:
(Overall comments, discuss strengths/weaknesses of the study, novelty, significance, general execution and scholarship, concerns about dual publication, research ethics, or publication ethics)

| **Reviewer** | **Comment** | **Response** |
| --- | --- | --- |
| **Reviewer #1:** | The article is of public health importance. However, future research is recommended with a larger sample for generalizability. | Thank you |
| **Reviewer #2:** | No Response |  |
| **Reviewer #4:** | The authors have done great work to revise the manuscript it now reads well. However, the responses to the above-stated comments, especially how they link their results in the discussion section of the paper would help in deciding on the manuscript. | Efforts have been made to address all the comments from the reviewers as required. We hope that the review has contributed to the improvement of the manuscript. |

Additionally, line numbers have changed due to the addition of recommended parts and the re-arrangement of the document.

A track-changed version has been prepared and can be attached to this resubmission.

A clean final copy has also been prepared; where possible, retrimmed to accommodate the required shortened sections as recommended, but precautions have been taken to avoid diverging the original meaning of sentences.

Yours sincerely

Charles E. Makasi
